# ‘Talking a different language’: an exploration of the influence of organizational cultures and working practices on transition from child to adult mental health services

**DOI:** 10.1186/1472-6963-13-254

**Published:** 2013-07-03

**Authors:** Susan McLaren, Ruth Belling, Moli Paul, Tamsin Ford, Tami Kramer, Tim Weaver, Kimberly Hovish, Zoebia Islam, Sarah White, Swaran P Singh

**Affiliations:** 1 Faculty of Health and Social Care, London South Bank University, London, United Kingdom; 2 Division of Mental Health and Well Being, Warwick Medical School, The University of Warwick, Coventry, United Kingdom; 3 Peninsula Medical School, University of Exeter, Exeter, United Kingdom; 4 Faculty of Medicine, Imperial College, London, United Kingdom; 5 Institute of Education, University of London, London, United Kingdom; 6 Research and Innovation Department, Birmingham and Solihull Mental Health Foundation Trust, Birmingham, United Kingdom; 7 St. George’s University of London, London, United Kingdom

**Keywords:** Transition, Culture, Working practices, Care continuity, Mental health

## Abstract

**Background:**

Organizational culture is manifest in patterns of behaviour underpinned by beliefs, values, attitudes and assumptions, which can influence working practices. Cultural factors and working practices have been suggested to influence the transition of young people moving from child to adult mental health services. Failure to manage and integrate transitional care effectively can lead to young people losing contact with health and social care systems, resulting in adverse effects on health, well-being and potential.

**Methods:**

The study aim was to identify the organisational factors which facilitate or impede transition of young people from child and adolescent mental health services (CAMHS) to adult mental health services (AMHS) from the perspective of health professionals and representatives of voluntary organisations. Specific objectives were (i) to explore organizational cultures, structures, processes and resources which influence transition from child to adult mental health services; (ii) identify factors which constitute barriers and facilitators to transition and continuity of care and (iii) make recommendations for service improvements. Within an exploratory, qualitative design thirty four semi-structured interviews were conducted with health and social care professionals working in CAMHS and AMHS in four NHS Mental Health Trusts and four voluntary organizations, in England.

**Results:**

A cultural divide appears to exist between CAMHS and AMHS, characterized by different beliefs, attitudes, mutual misperceptions and a lack of understanding of different service structures. This is exacerbated by working practices relating to communication and information transfer which could impact negatively on transition, relational, informational and cross boundary continuity of care. There is also evidence of a cultural shift, with some positive approaches to collaborative working across services and agencies, involving joint posts, parallel working, shared clinics and joint meetings.

**Conclusions:**

Cultural factors embodied in mutual misperceptions, attitudes, beliefs exist between CAMHS and AMHS. Working practices can exert either positive or negative effects on transition and continuity of care. Implementation of shared education and training, standardised approaches to record keeping and information transfer, supported by compatible IT resources are recommended, alongside management strategies which evaluate the achievement of outcomes related to transition and continuity of care.

## Background

Organisational culture is ‘that which is shared by members of groups and expressed in patterns of behaviour’ [1] and can be conceptualised as ‘shared values, beliefs, understandings and norms that bind organisational members into a collective endeavour’ [1,2]. Dimensions of organisational culture as embodied in approaches to cultural assessment can encompass leadership, organisational attributes, attitudes to change, safety, team orientation and working, collaboration, collegial and interdisciplinary relationships [3]. Culture is explicit in patterns of behaviour which can be reflected in working practices and rituals; beliefs, values, attitudes which justify behaviour patterns and are influential in decision making and assumptions based on beliefs, values and expectations [2,4]. Organisational culture is influential in shaping identities and role enactment by individuals [5]; in turn, professional roles, identities and working practices can create social boundaries which can hinder inter-professional relationships impeding the diffusion of innovation and change within and across organisations [6]. Recent interest has focussed on the possible relationship between organisational culture, performance (quality outcomes) and the potential for reshaping cultures as a lever for health care improvement. Currently, evidence supporting this is limited [2,7] and no convincing evidence of generalizable strategies to change culture have been found [8].

Although few studies have specifically investigated the influence of cultural factors and working practices, recognition that these could adversely influence the transition of young people from CAMHS to AMHS rests on variable sources of evidence relating to the evolution of separate child and adult mental health systems [9], complexity of organisational structures across which transfer takes place [10], differing provision of services [11-14], espousal of different models of health, illness and service delivery by CAMHS and AMHS [15,16] and working practices marked by lack of shared planning, poor communication and lack of formal arrangements to support transition [17-21]. Concerns have arisen that failure to manage and integrate care effectively during the process of transition can result in young people losing contact with health and social care systems, with adverse effects on health, well-being and potential [22-27].

Investigation of continuity of care in AMHS has highlighted emergent cultures in patterns of integrated, multi-disciplinary working by community mental health teams (CMHT) within and across organisational boundaries [28,29]. Integrated working in relation to communication, information transfer and decision making was a facilitator for cross-boundary, team and informational continuity of care. Barriers for cross boundary and team continuity were leadership styles, blurred professional roles, medical models of decision making, lack of training for role development and resources to support information transfer. Service users and carers identified both positive and negative ‘depersonalized transitions’ characterised in negative cases by poor communication and information transfer between services, users, carers and voluntary agencies [30].

Effective management of transition requires CAMHS and AMHS managers to face the challenge of implementing and monitoring uptake of standards and guidelines contained within national service frameworks, recent policy reviews and recommendations of professional bodies [31-34]. These encompass the appointment of key workers to co-ordinate the process, the development of transition protocols and care plans supported by joint strategic planning and multi-agency working aligned with the engagement of young people and families. Intrinsic to successful implementation and change management is an understanding of the influence of organizational cultures and working practices and if necessary, their reshaping through structural reform, to achieve aspirations for transition to be the ‘smooth process offering uninterrupted continuity of care with consideration of the young persons’ physical, social and psychological development [34]’. Given the limited evidence on cultural factors relating to transition, this paper presents findings from an exploratory study designed to illuminate cultures and working practices across the CAMHS/AMHS interface.

### Aim

The aim was to identify the organizational factors which facilitate or impede effective transition of young people from CAMHS to AMHS from the perspective of health professionals and representatives of voluntary organizations. Specific objectives were:

•To explore the organizational cultures, structures, processes and resources which could influence the transition from CAMHS to AMHS;

•To identify specific organizational factors which constitute barriers and facilitators to transition and continuity of care;

•To make recommendations for changes in services which could improve young people and carers’ experiences of transition and experienced continuity of care.

## Methods

This exploratory, qualitative study was conducted between 2007–2009, constituting the organisational strand (Phase 3) of the wider TRACK investigation, a multi-perspective, multi-site mixed methods study [35: Transition from CAMHS to AMHS: A Study of Service Organisation, Policies, Process, User and Carer Perspectives]. Wider objectives of TRACK included the conduct of a mapping study auditing policies and procedures relating to transition between NHS Mental Health CAMHS and AMHS; evaluation of the process of transition utilising a retrospective case note survey and exploration of the views of users, carers and mental health professionals on the process of transition. Findings of the organizational strand presented in this paper focus on two core themes of organisational culture, communication and working practices and nine related sub-themes, comprising the fifth in a series of papers reporting findings from the TRACK study [35-38]. The definition of continuity of care utilised in this paper is a multi-axial definition [39,40]; (Table 1).

**Table 1:** Defining continuity of care arr. Freeman et al. (2000) and (2002)

**Dimensions of continuity of care**	**Definition**
**Relational, personal, therapeutic continuity**	‘Providing one or more individual professionals with whom the service user can establish/maintain a consistent therapeutic relationship’
**Information continuity**	‘Effective communication based on excellent information transfer following the service user’
**Flexible continuity**	Adjusting to the needs of the individual over time
**Cross boundary, team continuity**	Effective co-ordination of services by teams/external agencies
**Long term continuity**	‘Provision of uninterrupted care as long as the service user requires it’
**Longitudinal continuity**	Care by ‘as few professionals as possible’ based on need

### Ethical approval

The study received ethical approval from Wandsworth Local Research Ethics Committee.

### UK mental health services configuration

In the United Kingdom, the National Health Service (NHS) provides a service free at the point of delivery financed through taxation. Mental health care for children and adolescents is delivered through a multi-agency model, encompassing community based Child and Adolescent Mental Health Services (CAMHS) delivering both medical and psycho-social care, together with a range of statutory and voluntary health and social care providers and education services. In terms of age cut-offs, CAMHS provide care up to 16–18 years and most AMHS have a lower age limit of 18 years [33]. AMHS are separate from CAMHS, but also work within a multiagency model, linked to other statutory and voluntary providers: both CAMHS and AMHS are separate from agencies responsible for housing support.

Within AMHS, health and social care are integrated, with continuity of care an important quality benchmark. In addition to acute in patient services, CMHT are intrinsic to the delivery of a multidisciplinary service to adult service users. Approaches to modelling community care have resulted in the inception of generic CMHT delivering a range of interventions to adults and specialist teams with specific remits for assertive outreach (difficult to engage service users), crisis resolution/ home treatment (avert hospitalisation in crisis) and early intervention teams (provide interventions for individuals 14–35 years with first presentation of psychotic symptoms).

### Sample

A total of thirty-four health and social care professionals working in four NHS Mental Health Trusts in Greater London and the Midlands and four staff each representing local voluntary sector organisations were recruited by RB (Tables 2 and 3) from February 2007 to April 2008. The rationale for including voluntary sector representation was their vital role in multi-agency working in mental health care, encompassing local provision of counselling services for young people and parents, school outreach services and parent support groups. The original intention was to purposively select equal numbers of staff from different professional groups representing CAMHS and AMHS from the two regions. Despite efforts to increase recruitment, (three follow-up mailings of invitations to participate) the intended recruitment target was not reached. In the final sample, there is under-representation from the Midlands and CAMHS are more strongly represented.

**Table 2:** Sample descriptors by sector and geographical location (n)

**Geographic area**	**Statutory sector**	**Voluntary sector**	**Total (n)**
**London**	20	2	22
**Midlands**	10	2	12
**Total (n)**	30	4	34

**Table 3:** Sample descriptors by service sectors and professional group (n)

	**Nurses**	**Social workers**	**Psychiatrists**	**Psychologists**	**Service Managers**	**Other sector**	**Total (n)**
**CAMHS**	5	3	4	2	2	-	16
**AMHS**	3	4	2	-	2	-	11
**CAMHS & AMHS**	-	-	-	-	3	-	3
**Voluntary sector**	-	-	-	-	-	4	4
**Total (n)**	8	7	6	2	7	4	34

### Semi-structured interviews

A semi-structured interview schedule was developed based on pilot fieldwork and review of the literature. Interviews of 40–60 minutes duration were conducted by telephone, arranged at a time convenient to the participant. Interviews were audio-recorded and transcriptions were independently checked for accuracy prior to analysis. Qualitative data analysis was conducted using a structured thematic approach [41] to systematically code, classify and organize interview content into key themes. Transcripts were read repeatedly by RB to identify recurring concepts and categories. These, together with issues incorporated within the interview topic guide, formed the basis of a conceptual thematic framework. This framework was used to code and classify data and modified and refined (by RB) throughout the analysis to reflect the content and issues expressed by respondents across the whole dataset. These coded categories and themes were then sorted and grouped into broader or higher-order themes (core themes) based on similarity of content (checked by SMcL).

Transcripts were imported to QSR Nu*Dist v.6.0 software to assist systematic and consistent coding and to identify patterns within the data suggesting possible differences in perceptions between Trusts, professional groups and/or CAMHS/AMHS. Illustrative quotes are provided to aid transparency of categorisation and theme representation (see Additional file 1: Core Theme: Service Cultures and Additional file 2: Core Theme: Communication and Working Practices). To protect anonymity, respondents are identified solely by professional group and whether working in CAMHS or AMHS. Trusts are not identified due to small numbers within some professional groups. Findings emanating from two core themes of service cultures, communication and working practices and nine related sub themes (Table 4) are reported below.

**Table 4:** Summary of core and sub-themes

**Core themes**	**Sub themes**
**1. Service Cultures**	Individual versus family approaches
	AHMS Lack of confidence with young people
	Impact of transition on parents and carers
**2. Communication and working practices**	Two-way communication and feedback Early communication
	Joint working and liaison
	Prior professional experience
	Inter-agency working practices and experiences
	Service user preparation for transition

## Results

### Core theme 1: Service cultures

A CAMHS Psychiatrist, CAMHS Trust Manager and voluntary sector participant all expressed the view that CAMHS and AMHS have different cultural approaches to service delivery, manifest in different beliefs, approaches, attitudes; the expression ‘talking a different language’ conveyed a powerful image (1a, 1b, 1c). CAMHS was perceived as more family-oriented, inclusive and holistic than adult services, which were perceived as focused more exclusively on the individual service user. CAMHS services were therefore seen as enabling a more proactive approach in engaging young people within their broader context of care. In contrast, AMHS were seen as crisis and medication intervention focused by the voluntary sector participant (1c). This raised issues about carers’ expectations of communication and level of involvement with AMHS, during and post-transition and how and by whom those expectations are managed.

However, whilst an AMHS nurse participant acknowledged a tendency not to engage with families (1e), both AMHS nurses and a psychologist stated that they considered family involvement, acknowledging the benefits of inclusion subject to the wishes of the young person (1d, 1e, 1f). One AMHS nurse also perceived that differences in professional background might impact on AMHS counterparts’ approaches to service users’ families and carers (1e). Ethical issues of confidentiality in relation to sharing of information were also raised by this participant. However, no reference was made to the use of ethical guidelines on sharing confidential information.

Psychiatrists from CAMHS and AMHS managers all perceived anxiety and lack of confidence in AMHS staff in managing young people which has important implications for continuing professional development and training (2a, 2b, 2c, 2d). From the perspective of one AMHS manager, this was partly explained by the shift toward post qualifying specialization, reducing staff confidence in their skills and abilities (2c). In contrast, the other AMHS manager suggested that AMHS staff possessed the skills to work with young people, but acknowledged a need for more generic training and practice aimed at anxiety reduction and restoration of confidence (2d).

There was also recognition, particularly by a CAMHS nurse (participant 2g), that the transition period from CAMHS to AMHS was difficult not only for young people, but also for parents. Psychiatrist and nurse CAMHS participants referred to the impact on parents and carers in terms of ‘loss’ or feeling ‘cut out (2e, 2f). As a consequence, from the perspective of a CAMHS nurse preparing families for the withdrawal of CAMHS input and with it a form of support they were unlikely to receive once a young person had left CAMHS needed to be carefully managed (2g).

### Core theme 2: Communication and working practices

CAMHS nurse and social worker participants identified a lack of two-way communication, whether verbal or written, with AMHS as a challenge affecting transitions (3a, 3b). A CAMHS psychologist explained that often administrators and gatekeepers to CMHTs were not co-located with the rest of the team, hindering effective communication (3c). Where communication was reciprocal it was perceived as facilitating transition by a CAMHS psychologist (3f). Another related challenge for communication between CAMHS and AMHS was seen by AMHS nurses to be different approaches to record keeping and care planning (3d, 3e). Different electronic record keeping systems between AMHS and CAMHS were cited as partly responsible, but also a lack of mutual understanding of formal Care Programme Approaches (CPA; [42]) processes more generally. Despite initial AMHS responsiveness in allocating young people to waiting lists, this could be seen as a hindrance to cross-service dialogue by a CAMHS psychologist (3f).

An AMHS manager and CAMHS nurse stressed the need for, and benefits of, early communication with regard to smooth and effective transition, allowing more time for the young person and their families to adjust to coming changes (4b, 4a). The importance of not leaving it too late to commence transition was also emphasized. An AMHS psychiatrist emphasised the importance of sharing information by all concerned in transition, together with a need for transparency on risks and care planning at handovers between professionals (4c).

Besides joint working (see below), three nurse participants from AMHS and CAMHS all agreed that staff having experience of working in the other service was very helpful in resolving day to day problems (4d, 4e, 4f). Having a shared professional identity with AMHS was helpful to two CAMHS nurse participants (4e, 4f) whilst prior knowledge and experience of the other teams working practices, information requirements, expectations and use of appropriate documentation was also found to be helpful to transition by CAMHS nurse (4f).

Psychiatrists from AMHS (5a) and CAMHS (5b) both described the benefits of having joint posts with a remit to enhance transition seamlessly between CAMHS and AMHS. However, the latter described how staff turnover, funding delays and cuts had resulted in loss of one post with a negative impact on transition (5b). Other approaches to joint working and liaison which facilitated transition included formal meeting arrangements between CAMHS and AMHS staff identified by a CAMHS nurse (5c) and from a CAMHS management perspective, AMHS psychiatrists holding clinics to facilitate joint assessments with CAMHS colleagues (5e). Proactive approaches by CAMHS staff to foster liaison with a range of mental health and learning disability teams identified as helpful by an AMHS manager (5d).

Shared responsibilities between AMHS and CAMHS care co-ordinators for a period of 3 months were found to be helpful in facilitating transition for the young person by a CAMHS social worker (6a), whilst a CAMHS manager and psychologist suggested a need not only for more shared meetings with AMHS, but the inception of joint training (6b, 6c).

A CAMHS psychiatrist (7a) and AMHS psychologist (7b) cited positive examples of inter-agency working with education services. A voluntary sector participant (7c) also described the positive benefit of CAMHS having had an educational psychologist seconded from the education service to work with parents and children in managing children and young people with Attention Deficit Hyperactivity Disorder (ADHD).

A CAMHS psychologist (8a) and AMHS nurse (8b) both recognised the challenges for young people encountering transition which, as noted by a CAMHS social worker (8e) and a CAMHS psychiatrist (8f) could lead to resistance to moving to AMHS. Most participants indicated a willingness to improve preparation of service users for transition, with some attempts being more successful than others; AMHS nurses (8b and 8c) noted the importance of clear accountability for care co-ordination and realistic expectations of AMHS provision. An AMHS manager (8c) identified the value of early involvement of parents and carers in visiting in-patient AMHS facilities, in reducing anxiety about transfer. Acknowledging the daunting nature of attending CPA meetings (8b), a CAMHS psychiatrist (8f) and psychologist (8g) also agreed that it was helpful for a CAMHS worker familiar to the young person to attend initial meetings with AMHS.

## Discussion

This study aimed to explore the influence of cultural factors and working practices on a collaborative endeavour, the process of transition for young people moving from CAMHS to AMHS and the potential impact on continuity of care. Findings have provided some evidence for a cultural divide in terms of different attitudes, beliefs, understanding and misperceptions between CAMHS and AMHS. Positive and negative working practices which could impact on continuity of care and transition were also identified, with reference to a multiaxial definition of continuity [39,40]. It is evident in these findings that factors other than cultural differences can impact on working practices, transition and continuity of care. Lack of resources, notably in relation to staff transition posts and IT provision were influential and other findings of TRACK identified wider staff shortages and heavy caseloads as problematic for organizational working [43]. It should also be borne in mind that the nature of the boundary between organisations and services can involve cultural, geographical, structural and financial dimensions, all of which can influence collaborative working and other outcomes.

A number of limitations are also acknowledged: these include under-representation in the sample of Trusts from the Midlands region and AMHS. The sample was drawn solely from health professionals and representatives of voluntary organizations; wider findings of TRACK, investigated experiences of service users, parents and other factors influencing transition [36-38].

The existence of a cultural divide in terms of attitudes, beliefs, understanding and some mutual perceptions between CAMHS and AMHS was evident: CAMHS was characterised (by CAMHS) as more person-centred, family oriented, positively focussed in terms of encouraging user resilience and utilizing talking therapies, but ‘talking a different language’ from AMHS. The latter was perceived by others as not such a good service, with a focus on crisis and medication management, problematic attitudes and a skill deficit underpinning staff lack of confidence in caring for young people. These findings extend those of others which have identified cultural differences present at the CAMHS/AMHS interface [15-17] and resonate with other TRACK findings relating to users and carers experiences, that AMHS care was medication oriented, psychiatrists dealt with medication but not emotional issues and that enduring mental health problems and taking medication were significant predictors of transition [36].

Indicators of more positive attitudes were evident in the acknowledgement by some AMHS staff of the importance of family involvement, notwithstanding the need to maintain confidentiality, which other studies have indicated can create an ethical dilemma for inter-professional working [44]. Other TRACK findings [38] established that many young people reported less involvement in care by parents and welcomed the new, post-transition confidential arrangements with AMHS. Given the different understandings, beliefs, mutual misperceptions, problematic attitudes and acknowledgement of skills deficits, a way forward could be to invest in common multidisciplinary training in adolescent health and transitional care, an approach to continuing professional development which has been advocated in guidance issued by policy makers. professional bodies and others [20,24,33]. Within a joint educational setting, CAMHS and AMHS professionals could explore cultural values, beliefs and assumptions and achieve a better understanding of their shared endeavour of promoting safe transition and continuity.

In relation to communication and working practices across the CAMHS/AMHS interface, findings revealed a mutual lack of understanding about service structures, location of key personnel and communication. The use of waiting lists was a source of frustration if it hindered further dialogue and different approaches to CPA management, documentation and electronic information transfer were evident. These findings are congruent with others [21] which have identified variable patterns of communication, limited opportunities for joint discussions between services and incompatibility of software systems as problematic, impacting negatively on transition and both information and cross boundary continuity of care [28,29]. A need to address the delay in national IT systems has been acknowledged [45] and clear accountability for key worker co-ordination of information transfer within care plans is advocated in policy guidance [33]. Enhancement of cross boundary and team continuity by providing training in communication skills as part of a wider educational programme on transitional care should also be implemented.

The challenges faced by young people, parents and carers prior to and during transition were understood by both CAMHS and AMHS staff and the potential for a disruption to established therapeutic relationships, impacting negatively on personal and relational continuity of care has been recognised more widely [29,30,33]. Feelings of ‘loss’, being ‘cut out’ of the system and ‘cultural shock’ were identified alongside anxieties evoked by feelings of intimidation at having to attend CPA meetings and meet new caseworkers; resistance to transfer could also be a problem for young people who were reluctant to embrace change. This professional view accords with experiences of parents post-transition who described less involvement in their childs’ care and had difficulties with readjustment. However some young people welcomed change and described positive experiences of gradual preparation for transition backed up by joint CAMHS/AMHS working and continuity of therapeutic relationships [38]. The importance of establishing early joint working to prepare young people and families for transition has also been acknowledged in many key reports [31-34] and the point made that when children enter a service, they should know from the outset when they can expect to leave it [20]. Concerns were expressed in this study about leaving it too late to initiate the transition process and it is interesting that none of our participants referred to the transition protocols, care pathways and care plans advocated in policy and professional body guidance intended to facilitate relational, informational and cross boundary continuity [24,25,31-34]. Other TRACK findings confirmed that protocols had been developed but were variable in their content and patterns of use [37]. No protocol specified how young people should be prepared for transition or detailed procedures to maintain continuity of care for those not accepted by AMHS. This is sometimes referred to as the policy-practice gap and raised questions about protocol utility and monitoring implementation in practice. Alternative models of transitional care, designed to overcome the policy-practice gap inherent in a protocol based approach have been suggested, ie the ‘Shared Management Framework’ focused on a transitions team and transitions co-ordinator [46].

A number of working practices were identified which were supportive of smoother and more efficient transition, enhancing cross boundary, information, personal and relational continuity of care and consistent with policy guidance [31-34]. These included the appointment of transition workers who worked across service boundaries, facilitating decision making on referrals, working with new care co-ordinators in AMHS for some time, joint appointments between services, shared meetings, co-working by psychiatrists and periods of parallel working by care co-ordinators. An interesting finding was that where staff had a shared professional identity and previous experience of either working in or managing both services, this was helpful in understanding working cultures and fostering joint working. Other very positive, proactive initiatives had been implemented, including the arrangement of pre-transition visits to AMHS by young people and families, working across organisational boundaries to foster joint working with external agencies and service providers, notably educational services, learning disability teams and family health liaison services; some of the managers who participated in this study had joint responsibilities for managing CAMHS and AMHS which could have facilitated these initiatives.

With emerging evidence that the bulk of adult mental health problems begin at a young age, the current CAMHS AMHS divide has produced a care pathway with maximum weakness where it should be most robust [47]. A radical redesign of youth mental health services has been suggested, but may be difficult to achieve in the short-term. Meanwhile it is imperative on services to improve transitional care and address the organisational factors that produce suboptimal care [36].

## Conclusions

Findings have demonstrated evidence of a cultural divide between CAMHS and AMHS, marked by different beliefs, attitudes, understanding and some negative misperceptions. Some working practices could constitute barriers to effective transition, relational, informational and cross boundary continuity of care. However, indications of a shift in bridging the cultural divide were also evident in positive approaches to collaborative working between services and external agencies which can support transition and continuity. Recommendations are that shared, multidisciplinary, continuing professional development should be implemented which is linked to appraisal and competency frameworks and focuses on transitional care. In context, this should encompass updating on service structures, utilisation of protocols, models of ‘best practice’ in transition, multi-agency working, communication skills, networking, and the acquisition of skills in working with young people and carers. Improvements in information transfer could be achieved by standardised approaches to record keeping supported by compatible IT resources.

Bearing in mind the growing independence of young people, family/carer involvement in transition needs to be sensitively negotiated between professionals, young people and families/carers. In the absence of generalizable strategies to foster cultural change [8] it is recommended that managers develop and implement service evaluation frameworks which identify and monitor outcomes of transition and continuity longitudinally. Combined strategies to implement change based on leadership, education and consistent application of guidelines and protocols are also recommended [48-50].

### RATS guidelines

This study meets the requirements of the RATS guidelines for the conduct of qualitative research.

## Competing interests

The authors declare that they have no competing interests.

## Authors’ contributions

SMcL and RB collected analysed and interpreted data. SMcL drafted the paper and RB, SPS, MP, TF, TK, TW, KH, ZI, SW contributed revisions to content and approved the final version. All authors contributed to the conception and design of the wider TRACK study (Principal Investigator Professor Swaran P Singh). All authors read and approved the final manuscript.

## Pre-publication history

The pre-publication history for this paper can be accessed here:

http://www.biomedcentral.com/1472-6963/13/254/prepub

## Supplementary Material

Additional file 1 Core Theme: Service Cultures. Click here for file

Additional file 2 Core Theme: Communication and Working Practices. Click here for file

## References

[B1] Alvesson M Cultural Perspectives on Organisations 1995 Cambridge: Cambridge University Press

[B2] Scott T Mannion R Marshall M Davies H Does organisational culture influence health care performance? A review of the evidence J Health Serv Res Policy 2003 8 2 105 117 10.1258/135581903321466085 12820673

[B3] Jung T Scott T Davies H Bower P Whalley D McNally R Mannion R Instruments for exploring organisational culture: a review of the literature Public Administration Review 2009 69 6 1087 1096 10.1111/j.1540-6210.2009.02066.x

[B4] Schein E Organisational Culture and Leadership 1995 San Francisco: Jossey-Bass

[B5] Fitzgerald L Cerian L Ferlie E Addicot R McGiven R Buchanan D Managing Change and Role Enactment in the Professionalised Organisation. Report to the National Co-ordinating Centre for NHS Service Delivery and Organisation (NCCSDO) 2006 London: NCCSDO

[B6] Ferlie E Fitzgerald L Wood M Hawkins C The non-spread of innovations: the mediating roles of professionals Acad Manage J 2005 48 1 117 134 10.5465/AMJ.2005.15993150

[B7] Mannion R Konteh FH Davies H Assessing organisational culture for safety improvement: a national survey of tools and tool use Qual Saf Health Care 2009 18 2 153 156 10.1136/qshc.2007.024075 19342532

[B8] Parmelli E Flodgren G Bryer F Baillie N Schafsma M Eccles M The effectiveness of strategies to change organisational culture to improve healthcare performance: a systematic review Implementation Science 2009 6 33 21457579 10.1186/1748-5908-6-33 PMC3080823

[B9] Davis M Sondheimer DL State child mental health efforts to support youth in transition to adulthood The Journal of Behavioural Health Services Research 2005 32 1 27 42 10.1007/BF02287326 15632796

[B10] Treasure J Schmidt U Hugo P Mind the gap: transition and interface problems for patients with eating disorders Br J Psychiatry 2005 167 398 400 16260812 10.1192/bjp.187.5.398

[B11] Davis M Geller JL Hunt B Within-state availability of transition to adulthood services for youths with serious mental health conditions Psychiatr Serv 2006 57 11 1594 1599 10.1176/appi.ps.57.11.1594 17085607

[B12] Audit Commission Children in Mind 1999 London: Audit Commission

[B13] Select Committee on Health Transitions Between Child/Adolescent and Adult Services Fourth Report. Provision of NHS Mental Health Services 2000 London: HMSO

[B14] Singh SP Evans N Sireling L Stuart H Mind the gap: the interface between child and adult mental health services Psychiatric Bulletin 2005 29 292 294 10.1192/pb.29.8.292

[B15] Singh SP Running an effective community mental health team Advances in Psychiatric Treatment 2000 6 414 422 10.1192/apt.6.6.414

[B16] Kipps S Bahu T Ong K Ackland FM Brown RS Fox CT Current methods of transfer of young people with type 1 diabetes to adult services Diabet Med 2002 19 649 654 10.1046/j.1464-5491.2002.00757.x 12147145

[B17] Lotstein DS McPherson M Strickland B Transition planning for youth with special health with special health needs: results from a national survey of children with special health needs Pediatrics 2000 115 1562 1568 15930217 10.1542/peds.2004-1262

[B18] Maitra B Jolley A Reder P, McLure M, Jolley A Liaison between child and adult psychiatric services Family Matters: Interface Between Child and Adult Mental Health 2000 London: Routledge 285 302

[B19] McDonagh J Kelly DA Transiting care of the paediatric recipient to adult care givers Paediatric Clinics of North America 2003 50 1561 1583 10.1016/S0031-3955(03)00131-7 14710793

[B20] McDonagh J Viner R Lost in transition? Between paediatric and adult services Br Med J 2006 332 435 436 10.1136/bmj.332.7539.435 16497736 PMC1382525

[B21] Richards M Vostanis P Interprofessional perspectives on transitional mental health services for young people aged 16–19 years J Interprof Care 2004 18 2 115 128 10.1080/13561820410001686882 15203671

[B22] American Academy of Paediatrics, American Academy of Family Physicians, American College of Physicians-American Society of Internal Medicine A consensus statement on health care transitions for young adults with special health care needs Paediatrics 2002 110 1304 1306 12456949

[B23] Kennedy A Sloman F Douglass JA Sawyer SM Young people with chronic illness: the approach to transition Intern Med 2007 37 8 555 560 10.1111/j.1445-5994.2007.01440.x 17640188

[B24] Royal College of Nursing Adolescent transition care 2003 London: RCN Publications

[B25] Royal College of Paediatrics and Child Health Bridging the Gap: Healthcare for Adolescents 2003 London: RCPCH Publications

[B26] Forbes A While A Ullman R Lewis S Mathes L Griffiths P A multi-method review to identify components of practice which may promote continuity in the transition from child to adult care for young people with chronic illness or disability 2001 London: Report for the NCCSDO

[B27] While A Forbes R Ullman S Lewis S Mathes L Griffiths P Good practices that address transition from child to adult care: synthesis of the evidence Child Care Health Dev 2004 30 439 452 10.1111/j.1365-2214.2004.00440.x 15320921

[B28] Burns T Catty J Clement S Harvey K Rees-Jones I McLaren S Rose D White S Wykes T Experiences of continuity of care and health and social care outcomes: the ECHO study 2007 London: Report for the NCCSDO

[B29] Belling R Whittock M McLaren S Burns T Catty J Rees-Jones I Rose D Wykes T Achieving continuity of care: facilitators and barriers in community mental health teams Implementation Science 2011 6 23 10.1186/1748-5908-6-23 21418579 PMC3073925

[B30] Rees-Jones I Ahmed N Catty J McLaren S Rose D Wykes T Burns T Illness careers and continuity of care in mental health services: a qualitative study of users and carers Soc Sci Med 2009 69 632 639 10.1016/j.socscimed.2009.06.015 19577834

[B31] Department of Health National Service Framework for Children, Young People and Maternity Services 2004 London: Department of Health

[B32] Department of Health Good transitions for good people 2006 London: Department of Health

[B33] A Good Practice Guide for Health Professionals and their Partners on Transition Planning for Young People with Complex Health Needs or a Disability 2008 London: Department of Health

[B34] Lamb C Hall D Kelvin R Van Beinum M Working at the CAMHS/Adult Interface: Good Practice Guidelines for the Provision of Psychiatric Services to Adolescents/Young Adults 2008 Joint Paper: Interfaculty Working Group. London. Royal College of Psychiatrists

[B35] Singh SP Paul M Islam Z Weaver T Kramer T McLaren S Belling R Ford T White S Hovish K Harley K Transition from CAMHS to Adult Mental Health Services: A Study of Service Organisation, Policies, Process, User and Carer Perspectives 2009 London: NETSCC SDO Project Reference 08/1613/117

[B36] Singh SP Paul M Ford T Kramer T Weaver T McLaren S Hovish K Islam Z Belling R White S Process, outcome and experience of transition from child to adult mental healthcare: a multi-perspective study Br J Psychiatry 2010 197 305 312 10.1192/bjp.bp.109.075135 20884954

[B37] Singh S Paul M Ford T Kramer T Weaver T Transitions of care from child and adolescent mental health services to adult mental health services (TRACK Study): a study of protocols in greater London BMC Health Serv Res 2008 8 135 142 10.1186/1472-6963-8-135 18573214 PMC2442433

[B38] Hovish K Weaver T Islam Z Paul M Singh S Transition experiences of mental health service users, parents and professionals in the United Kingdom: a qualitative study Psychiatr Rehabil J 2012 35 3 251 257 22246124 10.2975/35.3.2012.251.257

[B39] Freeman G Sheppard S Robinson I Ehrich K Richards S Continuity of Care: Report of a Scoping Exercise for the NCCSDO Programme 2000 London: NCCSDO

[B40] Freeman G Weaver T Low J Jonge E Crawford M Promoting Continuity of Care for people with severe mental illness whose needs span primary, secondary and social care: a multi-method investigation of relevant mechanisms and contexts 2002 NCCSDO: Report for the NCCSDO. London

[B41] Ritchie J Spencer L Bryman A, Burgess R Qualitative data analysis for applied policy research Analysing Qualitative Data 1994 London: Routledge 172 194

[B42] Department of Health Making the Care Programme Approach Work for You 2008 London: Department of Health http://webarchive.nationalarchives.gov.uk/+/www.dh.gov.uk/en/publicationsandstatistics/publications/dh_083650

[B43] Belling R McLaren S Paul M Ford T Kramer T Weaver T Hovish K Islam Z White S Singh SP Effects of organisational resources and eligibility issues on transition from child and adolescent to adult mental health services J Health Serv Res & Policyin press 24700210 10.1177/1355819614527439

[B44] Whittington C Leathard A McLaren S Whittington M Ethics and Social Care: Political, organisational and interagency dimensions. Chapter 6: 83–96 Ethics: Contemporary Challenges in Health and Social Care 2007 Bristol: Policy Press

[B45] Department of Health NHS Care Records Service: Connecting for Health 2006 http://systems.hscic.gov.uk/

[B46] Vloet M Davidson S Capelli M We suffer from being lost: formulating policies to reclaim youth in mental health transitions Healthc Q 2011 14 32 38 24956424 10.12927/hcq.2011.22361

[B47] McGorry P The specialist youth mental health model: strengthening the weakest link in the public mental health system Medical Journal of Australia 2007 187 7 S53 S56 17908028 10.5694/j.1326-5377.2007.tb01338.x

[B48] Forsetlund L Bjørndal A Rashidian A Jamtvedt G O’Brien MA Wolf F Davis D Odgaard-Jensen J Oxman AD Continuing education meetings and workshops: effects on professional practice and health care outcomes Cochrane Database Syst Rev 2009 http://apps.who.int/rhl/reviews/CD003030.pdf 10.1002/14651858.CD003030.pub2 PMC7138253 19370580

[B49] Paterson K Henderson A Trivella A Educating for leadership: a programme designed to build a responsive health care culture J Nurs Manag 2006 18 1 78 83 20465732 10.1111/j.1365-2834.2009.01065.x

[B50] Gattellari DG Grimshaw J O’Brien MA Local opinion leaders: effects on professional practice and healthcare outcomes Cochrane Database Syst Rev 2006 Issue 4 Art. No CD 000125 10.1002/14651858.CD000125.pub5 PMC6589938 31232458

