# Supplementary material for: ‘Talking a different language’: an exploration of the influence of organizational cultures and working practices on transition from child to adult mental health services
Source: BMC Health Serv Res. 2013 Jul 3;13:254. doi: 10.1186/1472-6963-13-254 (PMC3707757; doi:10.1186/1472-6963-13-254)
Supplement: Additional file 1 — Core Theme: Service Cultures. [file 1472-6963-13-254-S1.doc]

**Additional File 1: Core Theme: Service Cultures**

Core Theme: Service Cultures

| ***Sub-Theme: Individual versus Family Approaches*** |
| --- |
| ***1a:*** *“One is that CAMHS work in a much more systemic way than adult services. They often don’t see the young person in the context of their family or community. Sometimes it feels like we’re talking a different language… We would never see the young person in isolation. They are always with the system around them. I think adult services do find that more challenging and therefore their whole approach to intervention, resilience and recovery, whatever recovery means for that individual, is different to a CAMHS service. I think we’re more positive. We look at how you can use the system to move the individual forward and looking for resilience in the individual.”* *(Psychiatrist, CAMHS)*  ***1b****: “A big deal for the services is that Adults is not perceived as such a good service as CAMHS. CAMHS - well I suppose it’s more person centred – it has a lower threshold, it involves the family more, it’s a sort of kinder service than the rough and tumble of adult mental health services, so that is tricky for people and carers when I have spoken to them about the transition – it’s a difficult one because you get less of a service and a different attitude towards people and that is problematic.” (Trust Manager CAMHS &AMHS)*  ***1c:*** *“CAMHS and adult services do seem to be very different. CAMHS seems much more gentle and supportive and very much focused on talking therapies, whereas adult services tend to be about dealing with absolute crises and dealing with medication as an intervention. I think if you’re used to being in one and then you’re having to move to the another that’s a very big shock for people.” (Voluntary Sector)*  ***1d:*** *“I always think that the families should be involved. Certainly, not if the client doesn’t want you to have much to do with their family, then that’s fine but I would always try to encourage the families to tell us what they know. I am a firm believer that you can’t view any service user in isolation from their family and their environment. Sometimes we do dismiss families or not ask them and we lose a valuable resource there.” (Nurse, AMHS)*  ***1e:*** *“I think sometimes that is harder for people who have a nursing background more than a social work background, they find it harder and tend to think this is my patient and it is confidential between us and therefore I shouldn’t discuss anything with the family. Particularly if the person is living in the family home, I don't know how you cannot include them, they spend a lot more time with the person than we’ll ever spend with them.” (Nurse, AMHS)*  ***1f:*** *“I suppose we do – and I suppose this should be true with CMHTs also – we do automatically work with the families, which is helpful in engaging the clients and helping the transfers because then we get the parents’ approval of us or at least the parents at least feel they know something about what we’re doing and they can encourage.”* *(Psychologist, AMHS)* |
| ***Sub-theme: AMHS Lack of Confidence With Young People*** |
| ***2a*** *“I think there is some difficulty in the way that some of our adult colleagues view adolescents and people don’t always feel skilled in managing this client group…”* *(Psychiatrist, CAMHS)*  ***2b:*** *“Sometimes, the high levels of anxieties in working with young people. They tend to think ‘children’ and ‘I don’t know anything about children’.”* *(Psychiatrist, CAMHS)*  ***2c:*** *“I’ve watched things become more and more specialist and had to spend more and more time dealing with relationships and the confidence, because people lose confidence. You have a generic social worker moves into mental health team and all of a sudden they don’t know how to deal with children – how does that happen – and the opposite way, so it’s reducing some of the fear of talking to children – anybody can talk to children but it needs to be at different levels and for adult workers there’s this whole issue of how you talk to children about mental illness, about their parent’s mental illness.”* *(Trust Manager, CAMHS & AMHS)*  ***2d:*** *“I think most of the staff have got the skills but because they are not used that often, when they are called on to use them they have real concerns and it is something we all fear, is getting it wrong with children, I think these are the issues for adult staff, under-training. Again I think it could be relatively generic and that would make it much easier for staff to deal with that particular age group.”* *(Trust Manager, AMHS)* |
| ***Sub-theme: Impact of Transition on Patients and Carers*** |
| ***2e:*** *“We spend a lot of time liaising with schools, with other agencies and then moving on to adult it is very much about the individual so there can be quite big loss issues in relation to the parents or family structure or the supportive system who has been working with the young person.”* *(Nurse, CAMHS)*  ***2f:*** *“…our concern also is that we’ve always included the family as part of the intervention whereas our colleagues would not necessarily, automatically do that and at 18 a young person, in many cases, just be treated as an adult not really seen as part of the broader network and that causes a number of problems, particularly for parents who are carers and they feel that they’ve been completely cut out of the system.”* *(Psychiatrist, CAMHS)*  ***2g:*** *“….surprisingly, often it’s not so much the young person that’s bothered about the hand over, it’s the family….. so it’s actually been about involving parents in the idea that you’re not going to be around for much longer and really talking about what that means and giving them the opportunity to think about the next steps…”* *(Nurse, CAMHS)* |
