# Supplementary material for: ‘Talking a different language’: an exploration of the influence of organizational cultures and working practices on transition from child to adult mental health services
Source: BMC Health Serv Res. 2013 Jul 3;13:254. doi: 10.1186/1472-6963-13-254 (PMC3707757; doi:10.1186/1472-6963-13-254)
Supplement: Additional file 2 — Core Theme: Communication and Working Practices. [file 1472-6963-13-254-S2.doc]

**Additional File 2: Core Theme: Communication and Working Practices**

Core Theme: Communication and Working Practices

| ***Sub-theme:* *Two-way Communication and Feedback*** |
| --- |
| ***3a:****“The impact of parenting on the family is very important and we try to make sure adult services are aware of particular issues, but it doesn’t seem to work the other way with us. In fact, I can’t think of any instance where we have been told, this person is known to us or where we have been contacted by someone in adult services working with somebody where they know there are young children involved.”* *(Nurse, CAMHS)*  ***3b:*** *“There is a lack of communications, nobody comes back to you and says I’ve not received this or I’ve not received that until sometimes afterwards and sometimes it’s the case where you have to chase up with them.”* *(Social Worker, CAMHS)*  ***3c:*** *“That has been my experience and you get the team administrator who might not be in the same office as that person so can’t even tell you where that person is or what time they are going to be back and so these are the bits and pieces that you need to find out, whether there are any queries that you might have, they are a lot more difficult when you are not able to speak to that person or even know where that person is so you can call back at a later time.’ (Psychologist, CAMHS).*  ***3d:*** *“I think it would probably be around communication, that all the workers involved have good communication and because we both keep notes very differently it is making sure that those, that kind of paperwork, goes with the service user because we do read past stuff and it is useful to have that as a backup because some people get really angry when they have to keep retelling their story so I think it is about communication and about having an understanding of one another’s services I think.” (Nurse, AMHS)*  ***3e:*** *“One quite big thing that’s come up for us is documentation because I think general adult teams and specialists have been running with computers and with the system XXXX for a long time and we’ve had to do CPAs for a long time and on the system, whereas with CAMHS it just doesn’t seem very consistent and their understanding of who should be on enhanced CPA for instance can be quite different to ours, so things like that can be a bit tricky at times…”* *(Nurse, AMHS)*  ***3f*** *“I think the adult mental health service in the main are pretty responsive and I find they rarely quibble for example about referrals. In the main if we make a referral they say yes, absolutely, we’ll allocate that or pop that on a waiting list, so they are fairly streamlined. Now that has its advantages but actually it also has some disadvantages because one never then engages in a dialogue with anyone about the case but I don't think it is as frustrating as it might be.”* *(Psychologist, CAMHS)* |
| ***Sub-theme: Early Communication*** |
| ***4a:*** *“I think it was early communication, it was having clarity about what we were asking for and for some people it was a referral letter, that was as great as the involvement was, a referral letter and asking for the adult team’s consideration or opinion….so I think about whether it is going to be an appropriate referral and how it is best managed but it is about trying to bring services in as early as possible to do the thinking.”* *(Nurse, CAMHS)*  ***4b:*** *“Again from a personal clinical point of view I think often we leave it too late, we wait until somebody is 17½ and say right, now we need to do transition. If someone has been in service longer it is something we should start thinking about early on to allow not only the client but to allow the family to build up new relations with new members of staff.”* *(Trust Manager, AMHS)*    ***4c:*** *“A shared set of symbols and assumptions, shared by the patient as well as the people on both ends of the giving and handing over. Of course, those assumptions also include shared notions of risks. If someone tried selling me little Barry and they had polished him and shone him as a really nice, sweet little kid who stopped taking drugs ages ago, and the word drugs isn’t specified and they don’t tell you that Barry has just come of out Borstal after stabbing his dealer, and actually he’s got a snort of cocaine in his pipe every night kind of thing, that is not going to go down so well. You need to share risks clearly as part of the treatment plan.”* *(Psychiatrist, AMHS)* |
| ***Sub-theme: Prior Experience of Professional Staff*** |
| ***4d:*** *“Their new service manager is someone we know from adult mental health services which has actually made a big difference in terms of any problems. That is not about the protocol, that is much more about the personality…”* *(Nurse, AMHS)*  ***4e:*** *“I was a psychiatric nurse with adults so I have an understanding because I’ve worked in that situation…”* *(Nurse, CAMHS)*  ***4f:*** *“I think again because I’ve worked in adult for many years, it’s been easier for me to get transfers going because I know what the adult teams need to hear, so it’s about pushing the right buttons, so for me it’s gone pretty smoothly because I know that they want to know about risks, that they want to know about the actual illness and medication, and they want the right bits of paperwork. I know that that’s the kind of thing that’s going to make a transition smoother. I know I was very welcomed in this team because of that knowledge, who helped everyone out to know how to do that and be heard. I just know what to say – if someone isn’t listening to me, I’ll know what to say to get them to listen, it’s cheating a bit really – although it’s not really as people need that service, but it means I can kind of get through some of the difficulties more easily.”* *(Nurse, CAMHS)* |
| ***Sub-theme: Joint Working and Liaison*** |
| ***5a:*** *“Two years ago we had a post, which was a joint post with the (early intervention team) and that job worked really well because the person belonged to both teams and transferring the patient from our service was taken much earlier and then transferring them seamlessly and staying with their Care Co-ordinator once transferred to the other part earlier on the psychosis service, so that worked beautifully and then somebody left and it took us 14 months to renegotiate with adult services because they were restructuring, having lost money, redeployment, and everything else and the cuts… so there was a huge delay… The strength was the joint post, definitely. It was smoother, quicker, efficient, because what happens now without that post is that the patient does not want to go to adult services so we have a lot of resistance, patient resistance about transfer.”* *(Psychiatrist, CAMHS)*  ***5b:*** *“I think there’s a lot to be said for having a worker that’s specifically trained to target younger people and address these issues. We do know that when you have a special lead within an organisation, rather than a separate organisation, first of all they can act as an honest broker, or maybe if they get a nurse or an OT whatever, who takes the lead in adolescent areas, who has the local, as it were, patch knowledge. That’s probably a good idea. We don’t give enough resource or thought to that. But I must say it’s a relatively small number and that person needn’t have it as their substantive occupation.”* *(Psychiatrist, AMHS)*  ***5c:*** *“One of the things I found particularly helpful and I think I’ve been in a slightly privileged position compared with the rest of the team is I do liaison with one of the community mental health teams and I meet with their duty worker, only once every three months, but it keeps both of us kind of in touch,…”* (Nurse, CAMHS)  ***5d:*** *“…CAMHS workers going into the disability teams, learning disabilities and physical disabilities… Family Mental Health Liaison Service and that is just two people working into my CMHTs, so that’s the transition post. And then there are the Adolescent Mental Health team working with our Early Intervention team – there are some transition issues there – and case working, and then also the Parental Mental Health team. That’s interesting because it’s not about the transition from CAMHS to Adults particularly, it’s about the link between Adults, Mental Health and Children & Families teams, but it’s CAMHS workers who are doing that linkage.”* (Trust Manager, CAMHS & AMHS)  ***5e:*** *“One of the adult consultants does a clinic here once a month, where CAMHS colleagues can look to go and see them, discuss the case, agree to do a joint assessment with them and where appropriate, take them up in adult services. And that’s worked out very successfully.”* (Trust Manager, CAMHS) |
| ***Sub-Theme: Joint Working and Liaison*** |
| ***6a:*** *‘… I understand that it is difficult to completely switch over to a new team and set of professionals, so we share responsibility between care coordinators and gradually they get to see more of us and we start building up that relationship, so that transition becomes much easier rather than abrupt and sudden. We do that for about three months. This period is also used to prepare the person for transition, weaning away from their old team.”* *(Social Worker, CAMHS)*  ***6b:*** *“More joint working or more understanding between CAMHS and AMHS. I’m thinking about how different things are, for instance in child protection. CAMHS focus is very much on protection of a child whereas AMHS is more about supporting the adult. We come at it from a different perspective.* *Maybe we need to be looking more at joint training and looking at the issues within the context of the family.”* *(Trust Manager, CAMHS)*  ***6c:*** *We don’t have any shared training opportunities, any shared meetings which I think is unfortunate.”* *(Psychologist, CAMHS)* |
| ***Sub-theme: Interagency Working Practices and Experiences*** |
| ***7a:*** *“We are just at the moment forging links with Education so that young people again with first episode psychosis can be – they can be alerted to the fact that the young person is going to need additional input if they don’t already have that and we just recently had a meeting with the Head of the Educational Psychology so again, that’s been quite a difficulty because of the delay in getting young people back into some vocational training can really delay their progress.”* *(Psychiatrist, CAMHS)*  ***7b:*** *“We have a link with (named college and individual), whose job is to support vulnerable people who are wanting to study and so people go along and meet with him and he can advise them on different courses they might like to do and give them support while they actually do the courses, so that’s really helpful.”* *(Psychologist, AMHS)*  ***7c:*** *“Through Parent Partnership, they helped us, and CAMHS, they’d taken on the services of an Educational Psychologist who was seconded from the education service to work with parents and children, in managing the children’s behaviour. They helped us to promote the group and we started from there.”* *(Voluntary Sector)* |
| ***Sub-Theme: Service User Preparation for Transition*** |
| ***8a:****“I think for some young people it must feel like quite an abrupt transition. It’s not a process, it’s an event which doesn’t feel very comfortable.”* (Psychologist, CAMHS)  ***8b:*** *“But it is a scary time. CPA meetings are there, but they can be quite intimidating for people, so talking them through the kinds of questions they might want to ask beforehand. It’s difficult if the CMHT member coming is paying lip service, they don’t expect to take on any responsibility or will have to go back to their manager, or they haven’t thought who would be the young person’s care co-ordinator. It’s a bit random really.”* (Nurse, AMHS)  ***8c:*** *“I suppose the few cases I have been involved in, parents and carers have been quite heavily involved anyway and have been given the opportunity to come and look at services, that’s one of the things that we’ve done when we have had a child that was likely to move up, particularly to in-patient services, we have allowed the parents to come in and look at the service, where they would be getting their services from. It is really standard stuff, it is about taking some of the fear out of adult services and getting people involved very early on and being flexible.”* (Trust Manager, AMHS)    ***8d:*** *“I would have expected the CAMHS team to have prepared them (service users) for it but sometimes I think they have an unrealistic expectation of what adult mental health services can provide and that just goes back to they don’t know what we do so how can they tell families…”* (Nurse, AMHS)  ***8e:*** *“I suppose there is resistance on the part of the young person not to want to move, they don’t know the new worker, the set up, they have to re-familiarise themselves with somebody brand new, different styles of working, different techniques…”* (Social Worker, CAMHS)  ***8f:*** *“Young people who have had some form of psychotic breakdown and have engaged with the CAMHS team, often they don’t want to move on. They know they’re going to get something different. Part of that is just the fear of the Trust Manager and having to meet new people, but neither CAMHS nor the adult service have really had long enough to be able to manage that. So probably the best thing we will often do is meet and have a joint meeting with the adult team and the family.”* (Psychiatrist, CAMHS)  ***8g*:** *“I’ve also attempted to go with a young person to their initial involvement with adult services as a kind of handover to try to increase the likelihood of attending, rather than just send a letter to colleagues. So the young person can see the handover happening for them and where I have done that, it has been quite helpful in maintaining their engagement with adult services.”* (Psychologist, CAMHS) |
